# Forecasting the Value for Money of Mobile Maternal Health Information Messages on Improving Utilization of Maternal and Child Health Services in Gauteng, South Africa: Cost-Effectiveness Analysis

**DOI:** 10.2196/mhealth.8185

**Published:** 2018-07-27

**Authors:** Amnesty LeFevre, Maria A Cabrera-Escobar, Diwakar Mohan, Jaran Eriksen, Debbie Rogers, Annie Neo Parsons, Iman Barre, Youngji Jo, Alain Labrique, Jesse Coleman

**Affiliations:** ^1^ Division of Epidemiology and Biostatistics School of Public Health and Family Medicine University of Cape Town Cape Town South Africa; ^2^ Department of International Health Johns Hopkins Bloomberg School of Public Health Johns Hopkins University Baltimore, MD United States; ^3^ Wits Reproductive Health and HIV Institute Faculty of Health Sciences University of the Witwatersrand Johannesburg South Africa; ^4^ Department of Public Health Sciences and Department of Laboratory Medicine Karolinska Institutet Stockholm Sweden; ^5^ Praekelt Foundation Johannesburg South Africa; ^6^ Jembi Health Systems Cape Town South Africa; ^7^ Department of Public Health Sciences Karolinska Institutet Stockholm Sweden

**Keywords:** mHealth, cost effectiveness, cost utility analysis, digital health

## Abstract

**Background:**

Limited evidence exists on the value for money of mHealth information programs in low resource settings.

**Objective:**

This study sought to model the incremental cost-effectiveness of gradually scaling up text messaging services to pregnant women throughout Gauteng province, South Africa from 2012 to 2017.

**Methods:**

Data collection occurred as part of a retrospective study in 6 health centers in Gauteng province. Stage-based short message service (SMS) text messages on maternal health were sent to pregnant women twice per week during pregnancy and continued until the infant’s first birthday. Program costs, incremental costs to users, and the health system costs for these women were measured along with changes in the utilization of antenatal care visits and childhood immunizations and compared with those from a control group of pregnant women who received no SMS text messages. Incremental changes in utilization were entered into the Lives Saved Tool and used to forecast lives saved and disability adjusted life years (DALYs) averted by scaling up program activities over 5 years to reach 60% of pregnant women across Gauteng province. Uncertainty was characterized using one-way and probabilistic uncertainty analyses.

**Results:**

Five-year program costs were estimated to be US $1.2 million, 17% of which were incurred by costs on program development and 31% on SMS text message delivery costs. Costs to users were US $1.66 to attend clinic-based services, nearly 90% of which was attributed to wages lost. Costs to the health system included provider time costs to register users (US $0.08) and to provide antenatal care (US $4.36) and postnatal care (US $3.08) services. Incremental costs per DALY averted from a societal perspective ranged from US $1985 in the first year of implementation to US $200 in the 5th year. At a willingness-to-pay threshold of US $2000, the project had a 40% probability of being cost-effective in year 1 versus 100% in all years thereafter.

**Conclusions:**

Study findings suggest that delivering SMS text messages on maternal health information to pregnant and postpartum women may be a cost-effective strategy for bolstering antenatal care and childhood immunizations, even at very small margins of coverage increases. Primary data obtained prospectively as part of more rigorous study designs are needed to validate modeled results.

## Introduction

The use of mobile and wireless technology for health (mHealth) [1] has the potential to address critical gaps in timely and appropriate care-seeking across the continuum of care from pregnancy to postpartum [2]. mHealth solutions that target pregnant women have been shown to increase the utilization of antenatal care (ANC), skilled birth attendance (SBA), and childhood immunization rates [3]. While the program strategies and types of mHealth solutions have varied, delivery of health information content to pregnant women using short message service (SMS) text messaging has been effective in bolstering service utilization in several settings.

In Zanzibar and Malawi, maternal SMS text messaging initiatives have demonstrated a significant effect on the utilization of health services and health outcomes. In Zanzibar, the Wired Mothers Program provided unidirectional SMS text messaging and direct two-way communication using a free call voucher system to provide reminders for ANC visits; gestational age-specific reproductive, maternal, newborn, and child health (RMNCH) information; and an emergency medical response system. Program activities were associated with an increase in ≥4 ANC visits (OR 2.39, 95% CI 1.03-5.55) [4], in SBA among urban women (OR 5.73, 95% CI, 1.51-21.81) [5], and reduced perinatal mortality (OR 0.50, 95% CI 0.27-0.90) [6]. In Malawi, the Chipatala Cha Pa Foni program used a toll-free hotline to provide health information and advice, as well as tips and reminders through SMS text messaging tailored to the client’s week of pregnancy or child’s age. Program activities were associated with improved RMNCH knowledge and behavior including increased utilization of ANC within the first trimester, increased bed-net use for pregnant women and children, and breastfeeding within 1 hour of birth [7].

Beyond these programs, the Mobile Alliance for Maternal Action (MAMA) program has provided stage-based maternal health information using SMS text messaging in Bangladesh, India, Nigeria, and South Africa and has supported content to projects in 54 countries globally. With the exception of formative findings from activities in Bangladesh [8], evidence on the effectiveness of MAMA is limited. Findings from a retrospective case-control study in South Africa suggest a significant increase in the uptake of ≥4 ANC visits (Adjusted OR 3.21, 95% CI 1.73-5.98) and comprehensive care, defined as ≥4 ANC visits and receiving all vaccinations at 1 year of age (Adjusted OR 3.2, 95% CI 1.63-6.31) [9,10].

The emergence of data suggesting that stage-based SMS text messages on maternal health information may yield improvements in utilization across the continuum of care, from pregnancy, delivery to postpartum, is promising [4-6]. Yet, little is known about the value for money of maternal SMS text messaging initiatives. Cost-effectiveness analyses (CEAs) aim to generate evidence on the costs and consequences of 2 or more alternatives [11]. CEAs may be used in conjunction with affordability analyses for informed decision-making on the appropriate and optimal allocation of finite resources [11]. In the context of digital health programs, the costs required to establish and support the technological components of the program, including architecture, SMS text messaging delivery, device, and other costs, when coupled with the rapid pace of technology turnover, raise questions about the long-term viability and value of these initiatives compared with alternative resource uses.

To complement efforts to determine the effectiveness of MAMA in South Africa, we modeled the incremental cost-effectiveness of gradually scaling up SMS text messaging services to pregnant women throughout Gauteng province, South Africa from 2012 to 2017. This model-based analysis is anticipated to provide an early estimate of the cost-effectiveness of MAMA and inform future efforts to prospectively monitor costs and consequences of maternal SMS text messaging programs in low- and middle-income countries.

## Methods

### Setting

In South Africa, 1 in 24 children die before their 5^th^ birthday; 25% due to undernutrition, 25% in the first 28 days of life, and >50% outside of the formal health sector [12]. South Africa has the greatest number of HIV cases globally, and an estimated 32% of maternal deaths are attributed to HIV [13]. With a population of 13.5 million, Gauteng is South Africa’s most populous province and comprises 24% of the country’s total population [14]. An estimated 29% of the pregnant women in the study area and 12% of the total population in Gauteng were HIV+ [15]. In 2015, HIV and tuberculosis (TB) accounted for 24% of deaths among women and 27% among men 25-64 years of age [16]. In addition to the high burden of HIV and TB, noncommunicable diseases (NCDs) remain a leading cause of mortality and were the cause of over 80% of all deaths in 2015 [16]. While the utilization of care during pregnancy is universal, institutional births are high (97% in 2008-2009) [17], 86% of women receive postnatal care (PNC) in the clinic within 6 days of birth [16], and nearly all children under 1 year of age receive full immunizations [16], significant gaps persist in the timeliness, continuity, and quality of care. During pregnancy, only 49% of women attend their first ANC clinic prior to 20 weeks [16]. HIV testing coverage is the lowest in the country, and at 23%, falls well below the national average of 32%. Finally, timely diagnosis and appropriate management for hypertension (prevalence of 36% in 2012) and other risk factors for NCDs remain poor.

### Program Description

The MAMA program was initiated in 2012 in South Africa to bolster the utilization of RMNCH services among pregnant and postpartum women by sending registered users stage-based SMS text messages twice per week during pregnancy and up to the infant’s first birthday [9]. Women attending ANC (n=5111) and PNC (n=4953) services in 6 health facilities from June 15, 2012 to August 19, 2014 were registered to receive bi-weekly MAMA SMS text messages during pregnancy and up to the child’s first birthday. Content included information on maternal and postpartum danger signs, nutrition, and care-seeking during pregnancy, delivery, and postpartum. To approximate enrollment trends among pregnant women at scale across Gauteng, we projected the number of registered users based on the monthly trends observed during the MAMA implementation. By year 5, program activities were projected to expand to include the enrollment of an estimated 60% of all pregnant women in Gauteng.

### Study Design and Sampling

Data on costs and effects were collected during exit interviews with women attending ANC services in 6 clinics in Johannesburg as part of a retrospective case-control study performed from October 2014 to June 2015. Among 608 eligible women, 356 appeared for requested face-to-face interviews. Of these women, 181 had been allocated to the intervention group and 175 to the comparison group (Multimedia appendix 1). Despite completing exit interviews, significant gaps in the recording of health information on health cards existed, and reliance, where possible, was based on patients’ recall of services received. Final analyses were performed on 87 women enrolled during pregnancy to receive MAMA messages and 90 women enrolled into the comparison arm. Women with incomplete data records, those who did not show up for follow-up interviews, or who were enrolled into MAMA during the postpartum period were excluded from the final analyses.

### Health Effects

Primary outcome measures assessed as part of the retrospective case-control study included attendance rates for ANC visits 1 through 4 and immunization rates at birth, 6, 10, 14 weeks, and 9 months after birth. We have also presented estimates of the proportion of children fully immunized and those who received comprehensive care (defined as at least 4 ANC visits [ANC4+] and full immunizations). Despite data limitations, findings from the retrospective case-control study suggested that women exposed to SMS text messages were more likely to attend at least the recommended 4 ANC visits (OR 3.21, 95% CI 1.73-5.98) and complete comprehensive care, (OR 3.2, 95% CI 1.63-6.31) than women not exposed [10]. Rates for individual immunizations were also observed to increase by 1%-6%. However, the high baseline rates of utilization coupled with the small number of study participants with complete data indicated that the study was not powered to detect whether these trends were significant. Nevertheless, the trends observed were entered into the Lives Saved Tool (LiST) [18] to generate an estimate of lives saved through project activities for each year of implementation and adjusted for increases in the number of registered users over the 5-year analytic time horizon. Lives saved were translated into DALYs averted based on a 3% discount rate, no age weighting, and using a life expectancy of 66 years for a South African woman [19].

### Costs

For this model-based analysis, economic costs were estimated from a societal perspective inclusive of program, health systems, and user costs for a 5-year analytic time horizon (2012-2017). Program costs were defined as the costs required to develop, start-up, and support ongoing implementation. These were captured using an ingredients approach based on program activities, drawing from financial records and informant interviews with project implementing partners (Wits RHI, Cell-Life, and Praekelt Foundation), and through observations of health care workers providing routine ANC and PNC services within facilities. Costs were further categorized into capital (costs with a life expectancy of >1 year) and recurrent costs, with the former annualized over the lifetime of the project or life span of the item as appropriate and discounted at 3%. Development and start-up phase costs were viewed as one-time activities and similarly annualized over the lifetime of the project.

Incremental costs to the health system sought to capture costs associated with registration and increases in utilization. These included provider time costs to register patients into MAMA, as well as to provide routine clinical services during pregnancy and postpartum, including immunizations. These costs were estimated based on informant interviews, with provider salaries drawn from PayScale.com, an online salary, benefits, and compensation information company, and verified by the human resources department of Wits RHI.

Costs to users included all out-of-pocket payments incurred for care or treatment-seeking, including direct costs associated with medical care (consultation fees, medicine/commodity costs) as well as costs for transport/treatment-seeking and indirect costs due to wages lost resulting from time spent seeking care or away from income-generating activities. These were measured through patient interviews in intervention and comparison arms and generalized to the sample population as rollout occurred.

### Data Analysis

Costs were adjusted to 2015 USD dollars using consumer price indices [20] and a foreign exchange rate of 15.40 [21]. Capital costs were annualized using a 3% discount rate and estimates of local life expectancies. Health effects were analyzed using bivariate and multivariable logistic regression. Parameter costs and effects were adjusted to the projected sample population for each of the 5 years of implementation. Incremental costs were divided by incremental health effects to generate a deterministic estimate of the incremental cost-effectiveness ratio (ICER), expressed as a cost per life saved and cost per DALY averted. To test for uncertainty, one-way and probabilistic sensitivity analyses were conducted. The latter was performed in Microsoft Excel using a Monte Carlo simulation with 1000 iterations per analysis. The resulting mean point estimate was obtained by dividing mean costs by mean effects. The 95% CI for the ICER is presented based on percentiles. A cost-effectiveness plane and cost-effectiveness acceptability curve were used to calculate the probability that the intervention would be cost-effective for each of the several standard thresholds of cost-effectiveness. Cost-effectiveness was ultimately determined according to thresholds set forth by the Commission for Macroeconomics and Health and World Health Organization, which stipulate that an intervention is “highly cost-effective” and “cost-effective” at 1 and 3 times, respectively, the value of per capita gross domestic product per DALY averted. To facilitate comparison with alternative resource uses, we additionally compared findings against those available in the literature, including the Disease Control Priorities Project 3^rd^ edition, which highlights low-cost high-priority interventions for key regions globally.

## Results

**Figure 1:**
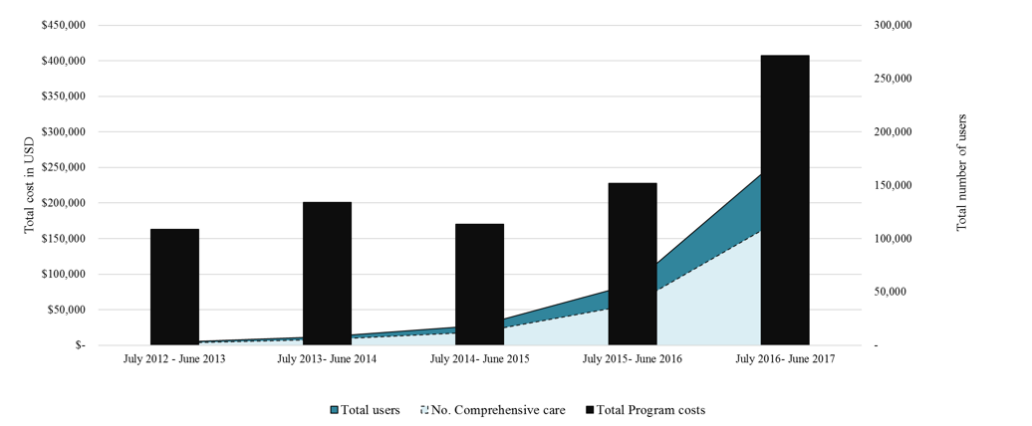
Observed and forecasted enrollment trends over 5 years: July 2012-June 2017. Total users denote women registered to receive MAMA messaging while comprehensive care refers to the subpopulation that attended all recommended antenatal care 1 to 4 visits and had children that received the fully package of immunizations.

Figure 1 presents data on the observed and forecasted enrollment trends over the 5-year analytic time horizon of the project. In years 1 and 2, a total of 2879 and 8161 women were enrolled to receive MAMA messages, representing 1% and 3% of total pregnancies in Gauteng, respectively. Extending this monthly pattern of enrollment, we estimated a 10% monthly growth in the number of users registered to the program. This corresponds to a total of 18,419 (6% of pregnant women), 57,214 (19% of pregnant women), and 179,562 (60% of pregnant women) enrolled in years 3, 4, and 5 of the program, respectively.

Table 1 presents program costs for implementation and technology support for each year of the program. Years 1 and 2 reflect costs incurred by the program, whereas years 3-5 represent forecasted costs anticipated with the scale up of implementation across Gauteng. Among key sub-categories of costs, technology support costs comprised 63% of total program costs, half of which was spent on SMS text message delivery costs. The annual estimates of implementation support costs were primarily attributed to the annualized estimates of development costs incurred at the project’s inception to support content development and localization and the development of training materials. Overall, personnel costs comprised 18% of annual costs, including 9% for program support staff and 9% for technology support and management personnel.

**Figure 2:**
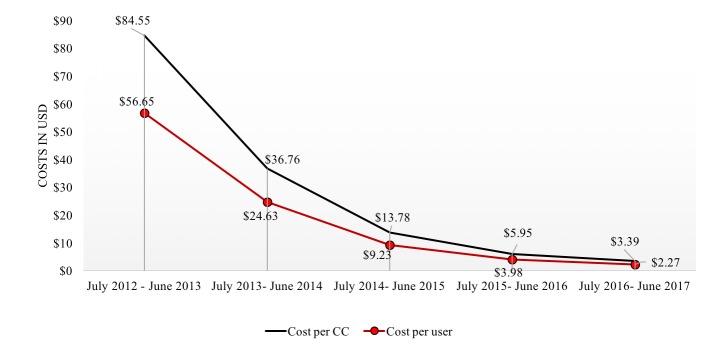
5-year trends in the total program cost per registered user and per case of comprehensive care (CC) received among MAMA users over 60 months.

Figure 2 presents data on trends in the total program cost per registered user and per case of comprehensive care (≥4 ANC and full immunization). Multimedia Appendix 2 summarizes year 5 parameter inputs for the probabilistic sensitivity analyses (similar tables are presented in Multimedia Appendices 3-6 for each of the first 4 years of the program). By year 5, out of 179,562 registered users across Gauteng, 95% of those exposed to MAMA were estimated to have received all childhood immunizations compared with 90% of non-MAMA users. Similarly, 72% of those exposed to MAMA received ≥4 ANC visits compared with only 46% in the comparison arm. A total of 67% of MAMA users were estimated to receive comprehensive care compared with 39% of non-MAMA users. By inputting individual coverage data into LiST, we estimated that a total of 182 (range, 109-199) lives would be saved in the year 5 of the program. Multimedia Appendix 7 presents data on the number of individuals expected to attend ≥4 ANC visits and have fully immunized children and the total number of comprehensive care users by year 5 of the program along with corresponding estimates of the lives saved and DALYs averted.

**Table 1:** Forecasted 5-year costs in 2015 US $ for gradual rollout in Gauteng province, South Africa.

Category		Total program cost, July 2012 to June 2013	Total program cost, July 2013 to June 2014	Forecasted, July 2014 to June 2015	Forecasted, July 2015 to June 2016	Forecasted, July 2016 to June 2017	Total cost over 5 years (% of total cost)	
**Implementation support**								
	Development	37,353.42	38,474.03	39,628.25	39,628.25	42,041.61	197,125.55 (17)	
	Start-up	17,765.76	18,298.74	18,847.70	18,847.70	19,995.52	93,755.41 (8)	
	Training	149.73	—	73.86	—	80.51	304.10 (0)	
	Personnel	18,375.80	18,798.00	19,630.41	20,514.67	21,398.94	98,717.82 (8)	
	Buildings	5974.80	5644.53	5894.48	6160.00	6425.52	30,099.33 (3)	
	Transport	3223.16	3044.99	3179.83	3323.06	3466.30	16,237.34 (1)	
	Communication	537.19	507.50	529.97	553.84	577.72	2706.22 (0)	
	Subtotal implementation support	83,379.87	84,767.78	86,081.31	89,027.53	93,986.12	437,242.60 (37)	
**Technology costs**								
	Start-up/development	129.89	129.89	137.03	144.08	158.74	699.63 (0)	
	Content maintenance	10,478.75	12,876.98	9167.21	9442.22	9725.49	51,690.64 (4)	
	Technology maintenance	8279.94	36,864.04	18,509.92	19,333.07	20,156.87	103,143.84 (9)	
	Project management/personnel	25,697.90	30,970.64	15,588.69	16,625.15	17,709.78	106,592.15 (9)	
	Monitoring and evaluation	1842.36	1961.43	108.72	111.99	115.34	4139.84 (0)	
	Building/overhead	10,073.21	13,088.45	12,225.42	12,765.21	13,305.66	61,457.94 (5)	
	Travel	11,353.19	8606.91	3100.02	3237.30	3374.71	29,672.13 (3)	
	SMS text message delivery	5384.11	9857.96	23,233.68	75,421.98	246,909.46	360,807.20 (31)	
	SMS text message translation	1736.80	1855.87	1855.87	1855.87	1855.87	9160.28 (1)	
	Printing	4726.26	—	—	—	—	4726.26 (0)	
	Subtotal technology	79,702.40	116,212.16	83,926.55	138,936.88	313,311.93	732,089.92 (63)	
Total		163,082.27	200,979.94	170,007.86	227,964.40	407,298.04	1,169,332.51 (100)	

Estimates of service utilization were used to forecast the costs to the health system associated with registering pregnant women to MAMA as well as treating additional cases (Multimedia Appendix 2). Assuming a 3-minute registration time, provider costs to register each woman were estimated to be US $0.08 (range of $0.04-0.11), while ANC counseling service delivery costs were estimated to be US $1.28 for ANC 1 and US $1.03 for each of the remaining ANC 2-4 visits. PNC was estimated to cost US $1.03 for PNC 1 and US $0.51 for each of the remaining 5 visits. Adjusting for differentials in utilization across study arms, total ANC 4+ and PNC 5+ costs were $1,101,947 in the MAMA study arm and $857,070 in the comparison arm.

Mean costs to users for attending ANC and PNC were drawn from structured interviews and included food, wages lost, child care, and transport costs (Multimedia Appendix 2). The mean cost per visit was $1.66 for ANC and $1.48 for PNC. The largest proportion of costs were attributed to wages lost (90%), followed by transportation (5%), child care (4%), and food (1%). Multimedia Appendix 3 summarizes data on costs and consequences for years 1-5 by study arm. Deterministic estimates of the incremental cost per live saved from a societal perspective ranged from US $56,011 in year 1 to US $5652 in year 5. Estimates of the cost per DALY averted similarly declined from US $1985 in year 1 to US $200 in year 5. Probabilistic estimates mirror this pattern.

**Figure 3:**
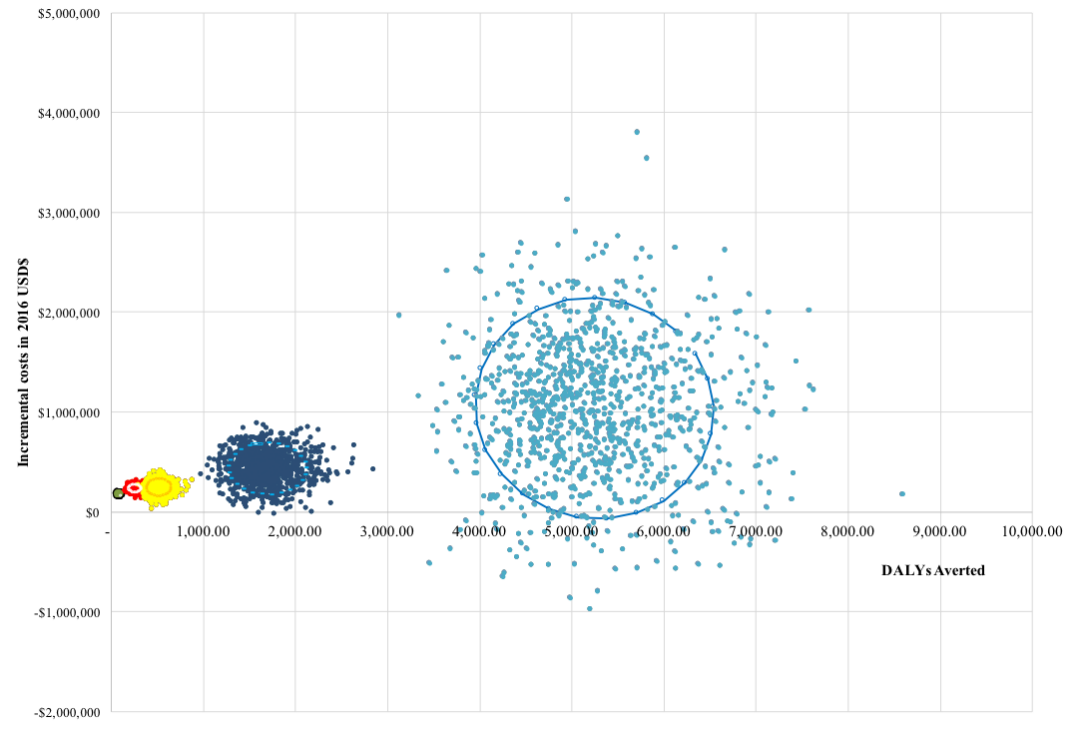
Cost effectiveness plane of years 1-5 of MAMA implementation vs. Status quo in Gauteng, South Africa. Individual dots represent the incremental costs and incremental disability adjusted life years (DALYs) averted for each of 1,000 simulations conducted by year of implementation.

**Figure 4:**
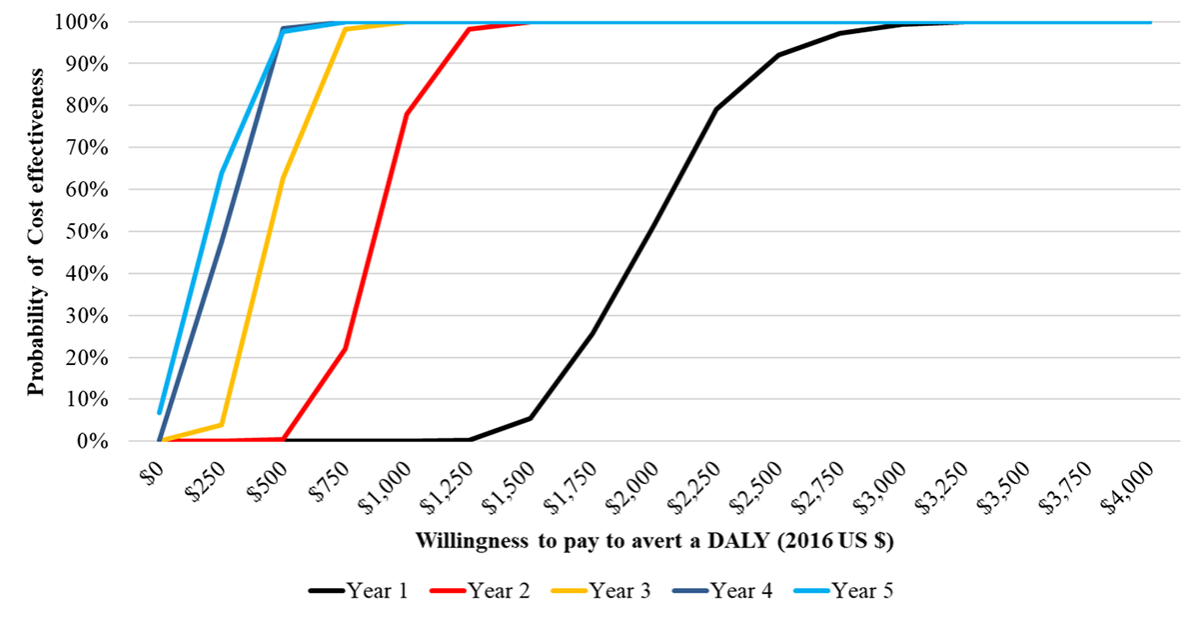
Incremental cost effectiveness acceptability curve of years 1-5 of MAMA implementation vs status quo in Gauteng, South Africa. Using the South Africa’s gross national income (GNI) per capita for 2015 of US $6,080 as the threshold, program activities have a 100% probability of being cost effective. At lower willingness pay thresholds, the probability of MAMA being cost effective increases over time as the number of users increases along with anticipated health effects. DALY: Disability adjusted life years.

Figure 3 presents the cost-effectiveness plane for each year, 1 through 5, whereas Figure 4 depicts incremental cost-effectiveness acceptability curves for each year of implementation. The cost per DALY averted falls beneath the 2015 Gross National Income (GNI) for South Africa of US $6080 for each of the 5 years of implementation. At a lower willingness-to-pay threshold of $250, the probability of achieving cost-effectiveness ranges from 0% in year 1 to 64% in year 5.

Using the South Africa’s Gross National Income per capita for 2015 of US$6080 as the threshold, program activities have a 100% probability of being cost-effective. At lower willingness-to-pay thresholds, the probability of MAMA being cost-effective increases over time as the number of users increases along with anticipated health effects.

**Figure 5:**
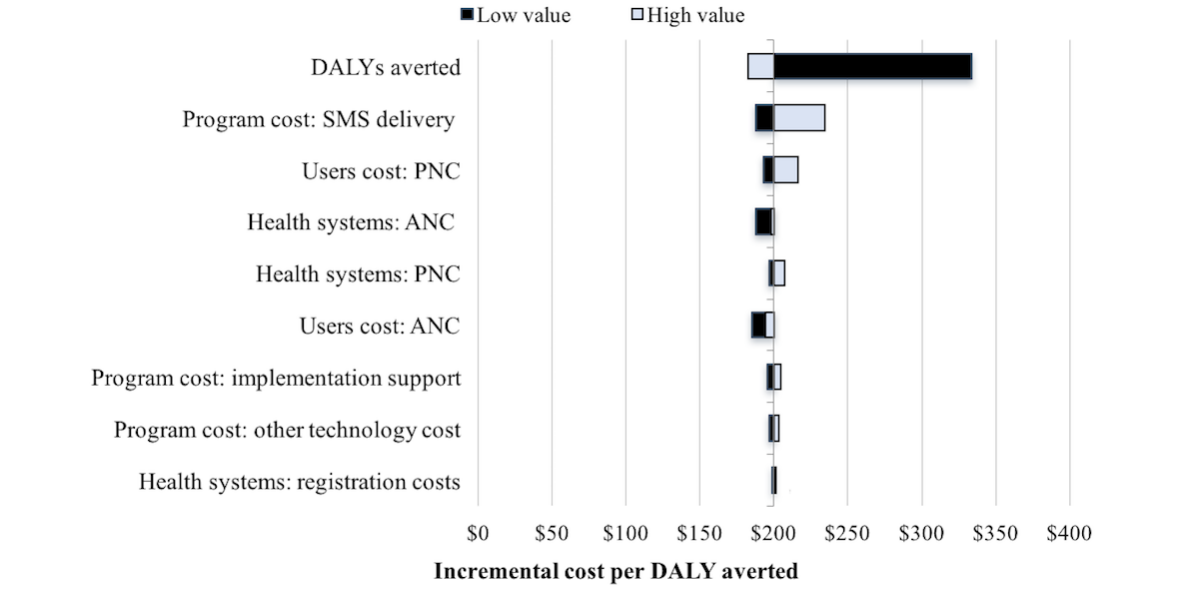
One way sensitivity analysis of key drivers of incremental cost per disability adjusted life year (DALY) averted for year 5 of program implementation (all costs in US $). ANC: antenatal care; PNC: postnatal care; SMS: short message service.

To compliment probabilistic sensitivity analyses, we also conducted one-way sensitivity analyses to identify key drivers of the incremental cost per DALY averted (Figure 5). The leading driver of incremental cost-effectiveness is the number of lives saved and corresponding number of DALYs averted, followed by programmatic costs associated with SMS text message delivery costs.

## Discussion

### Summary of Key Findings

Study findings modeling the incremental cost-effectiveness of exposure to SMS text messages during delivery and postpartum on care-seeking and childhood immunizations suggest that the cost per DALY averted ranges from US $1985 in year 1 when only 1% of pregnant women are registered to US $200 in year 5 when 60% of pregnant women are included. Societal costs to implement MAMA in the 5^th^ year of implementation were estimated to be US $3.6 million dollars, 59% of which represent costs borne by users to seek care for ANC and PNC, 30% costs to the health system, and 11% program costs. When considered against a status quo comparator, the incremental annual cost to implement MAMA at 60% coverage is US $1.03 million.

To estimate the health effects of SMS text messaging exposure, we drew from sample data on changes in the utilization of services among MAMA users and nearby non-MAMA-using mother-infant pairs for ANC and childhood immunizations up to 9 months. Less than 100 complete records were available in each study arm. When coupled with the existing high rates of service utilization, it meant that we were not powered to measure changes in the utilizations rates for individual vaccines. However, we were powered to measure observed changes in the utilization for ANC and comprehensive care (defined as ANC 1-4 and full immunizations). Based on available data, 1%-4% increases in immunization rates were observed by vaccine type along with a 14% increase in all 4 ANC visits. The latter finding is consistent with changes in coverage observed in Zanzibar as part of the Wired Mothers Program [4] and with emerging findings from the mCare project in Bangladesh. However, it is noteworthy that the overall utilization of ANC is higher in South Africa than elsewhere in the region, including Zanzibar; thus, it is feasible that we have overestimated health effects. Efforts to account for this were made through one-way and probabilistic sensitivity analyses, which sought to explore the effects of changing individual parameters, including coverage, on overall cost-effectiveness.

In the 2 years since the MAMA program ended, the National Department of Health (NDOH) has developed and rapidly scaled a maternal SMS text messaging program called “MomConnect,” which is based on MAMA but with less specific messages on the prevention of mother-to-child transmission of HIV. Like MAMA, MomConnect registers pregnancies and links expectant mothers to gestation age-specific pregnancy information, while also providing access to a help desk for reporting compliments or complaints on service delivery. Since its inception, MomConnect has grown to become one of the largest mHealth initiatives globally, registering >1 million pregnant women in >95% (3300) of health facilities in South Africa to receive SMS text messages on maternal health information [22]. While data on the cost-effectiveness of MomConnect are not available, trends in the number of registered users in this analysis mirror those attained. With many of the technology partners overlapping, it is feasible that costs and value for money estimates will be similar.

Elsewhere, data on the value for money of digital health programs are slowly emerging. However, to our knowledge, this is the first study to provide evidence on the value for money of maternal SMS text messaging programs. To date, a dozen peer-reviewed papers comprise the body of evidence on the value for money of mHealth solutions, including CEAs (6 studies) [23-26], cost-utility analyses (2 studies) [27,28], and cost-benefit analyses (4 studies) [29-32]. The distribution of digital health application types include 4 studies focused on client education and behavior change communication, 2 on electronic decision support, 2 on provider training and education, 2 on sensors and point-of-care diagnostics, and one on provider-to-provider communication [33]. Across disease areas, 42% of studies focus on general health, 25% on infectious diseases, 25% on chronic diseases, and 8% on women’s health [33]. Efforts to compare these studies with MAMA are challenging, given the differences in the outcome measures used. None of the identified CEAs report outcome metrics in units comparable (eg, lives saved) to those reported here, and only one of the 2 cost-utility analyses used DALYs as the outcome measure. Where the latter is concerned, a 2012 cost-utility analysis of a tuberculosis control strategy in Thailand, wherein patients received daily SMS text messages from a village health volunteer reminding them to take their medication, was associated with a median ICER of 350 international dollars (or US $4270) per DALY averted [27]. However, the uncertainty ranges around the health gain in DALYs were wide and crossed zero, suggesting that no distinction could be made in cost-effectiveness between mobile phone reminders and the comparator [27]. Findings from a 2013 cost-utility analysis of telemedicine to screen for diabetic retinopathy in India reported that screening once in a lifetime (US $2692 per Quality Adjusted Life Year, QALY, gained), twice in a lifetime (US $2475 per QALY gained), and every 5 years (US $3134 per QALY gained) were cost-effective using the WHO threshold recommendations [28]. However, annual screenings and/or those every 3 years did not fall below the threshold for cost-effectiveness [28]. Collective consideration of this body of evidence, in conjunction with our study findings, suggests that mobile SMS text messaging interventions for maternal health may compare favorably with the handful of other cost-effectiveness studies of mHealth interventions emerging from lower- and middle-income countries.

### Limitations

From the outset, we sought to base this analysis on primary data and focus only on the 2-year analytic time horizon of the program. However, primary data collection efforts were hampered by challenges in patient recruitment and data completeness. While we sought to obtain data on immunizations from the paper-based booklets provided by NDOH to mothers and completed by health workers at the time of service delivery, in practice, significant gaps in the completeness and quality of record keeping meant that a large proportion of interviewed participants were excluded from the final analyses. In shifting to a model-based analysis, we sought not only to more rigorously capture uncertainty but also consider the implications of service delivery at scale. Except for variable costs associated with SMS text message delivery, much of the technological costs associated with the MAMA program activities were fixed, irrespective of the scale of implementation. That said, our findings clearly suggest that greater value for money is attained with increasing scale. We hope that further analyses drawing from primary data of maternal SMS text messaging at scale through MomConnect and other initiatives will confirm this.

### Conclusions

This is a first of its kind study to provide an evidence-informed model of the value for money of maternal SMS text messaging programs. Study findings suggest that SMS text messages to pregnant and postpartum women are cost-effective, according to the GNI per capita thresholds for South Africa. Cost-effectiveness improves with scale. Further efforts are needed to determine the value for money of maternal SMS text messaging under more robust study designs and in differing settings where technological (network coverage and access to mobile phones), epidemiological, and health systems profiles may differ.

## Abbreviations

ANC: antenatal care
CC: comprehensive care
DALY: disability adjusted life year
GNI: gross national income
ICER: incremental cost-effectiveness ratio
LiST: Lives Saved Tool
MAMA: Mobile Alliance for Maternal Action
NDOH: National Department of Health
PNC: postnatal care
QALY: quality adjusted life year
RMNCH: reproductive, maternal, newborn, and child health
SBA: skilled birth attendance
SMS: short message service

## Appendix

Multimedia Appendix 1 Eligibility and enrollment flow of study participants included.

Multimedia Appendix 2 Parameters for probabilistic sensitivity analysis drawing from Year 5 costs (All currency in US $).

Multimedia Appendix 3 Year 1 Program costs in US $ for gradual rollout in Gauteng province, South Africa.

Multimedia Appendix 7 Year 2 Program costs in US $ for gradual rollout in Gauteng province, South Africa.

Multimedia Appendix 5 Year 3 Program costs in US $ for gradual rollout in Gauteng province, South Africa.

Multimedia Appendix 6 Year 4 Program costs in US $ for gradual rollout in Gauteng province, South Africa.
