# Supplementary material for: Forecasting the Value for Money of Mobile Maternal Health Information Messages on Improving Utilization of Maternal and Child Health Services in Gauteng, South Africa: Cost-Effectiveness Analysis
Source: JMIR Mhealth Uhealth. 2018 Jul 27;6(7):e153. doi: 10.2196/mhealth.8185 (PMC6086931; doi:10.2196/mhealth.8185)
Supplement: Multimedia Appendix 2 [file mhealth_v6i7e153_app2.pdf]

Parameters for probabilistic sensitivity analysis drawing from year 5 costs. (All currency in US $)

			MAMA[c]			Comparison			Incremental
Parameter			*Base case*	*High*	*Low*	*Base case*	*High*	*Low*	
Effects									
		Proportion ANC[d] 4+	72%	85%	55%	46%	53%	38%	26%
		Number ANC 4+ Gauteng	129,284	152,627	98,759	82,598	95,168	68,233	46,686
		Proportion Fully immunized	95%	98%	92%	90%	94%	84%	5%
		Number Fully immunized	170,584	175,970	165,197	161,605	168,788	150,832	8978
		Proportion ANC4+ & Fully immunized	67%	75%	57%	39%	53%	26%	
		Number ANC4+ & Fully immunized	120,306	134,671	102,350	70,029	95,168	46,686	50,277
		Inputs into Lives Saved Tool							
		ANC[a]	98%	99%	91%	84%	87%	82%	14%
		Bacillus Calmette–Guérin	99%	100%	86%	97%	99%	83%	2%
		Polio - 3 doses	99%	99%	86%	98%	99%	85%	1%
		Pentavalent	99%	99%	86%	97%	99%	78%	2%
		Pneumococcal - 3 doses	98%	99%	96%	94%	98%	82%	4%
		Measles- single doses	98%	99%	96%	94%	98%	82%	4%
		Incremental Lives Saved[b]							182 (109-199)
		Disability adjusted live years averted (3% discounting, no age weighting)							5145 (3080-5628)
Provider costs									
		Peer educator time costs to register MAMA users	$0.08	$0.11	$0.04	-	-	-	
Registration costs Gauteng			*$13,808.85*	*$20,188.24*	*$7747.75*				*$13,808.85*

		ANC 1 Group counseling (5 minute peer educator)	$0.26	$0.37	$0.14	$0.26	$0.37	$0.14	
		ANC 1 One on one consultation (10 minute Nurse time)	$1.03	$1.50	$0.58	$1.03	$1.50	$0.58	
		Total ANC1	*$1.28*	*$1.87*	*$0.72*	*$1.28*	*$1.87*	**$0.72**	
		ANC 2	$1.03	$1.50	$0.58	$1.03	$1.50	$0.58	
		ANC 3	$1.03	$1.50	$0.58	$1.03	$1.50	$0.58	
		ANC 4	$1.03	$1.50	$0.58	$1.03	$1.50	$0.58	
		Total ANC 4+	*$4.36*	*$6.37*	*$2.45*	*$4.36*	*$6.37*	**$2.45**	
ANC 4+ Gauteng			*$563,401.28*	*$629,200.02*	*$373,183.51*	*$359,950.82*	*$434,720.02*	*$232,690.89*	*$203,450.46*
		PNC[e] 1 (10 minute Nurse time)	$1.03	$1.50	$0.58	$1.03	$1.50	$0.58	
		PNC 2 (5 minute Nurse time)	$0.51	$0.75	$0.29	$0.51	$0.75	$0.29	
		PNC 3 (5 minute Nurse time)	$0.51	$0.75	$0.29	$0.51	$0.75	$0.29	
		PNC 4 (5 minute Nurse time)	$0.51	$0.75	$0.29	$0.51	$0.75	$0.29	
		PNC 5 (5 minute Nurse time)	$0.51	$0.75	$0.29	$0.51	$0.75	$0.29	
		Total PNC 5	*$3.08*	*$4.50*	*$1.73*	*$3.08*	*$4.50*	*$1.73*	
		PNC5+ (Fully immunized) Gauteng	*$524,736.48*	*$742,927.09*	*$303,711.97*	*$497,118.77*	*$678,324.73*	*$291,315.57*	*$27,617.71*
		Total provider cost per ANC4+ & Fully immunized Gauteng	*1,101,947*	*1,392,315*	*684,643*	*857,070*	*1,113,045*	*524,006*	*$244,877.03*
Users' costs									
	Mean cost per person per visit								
		Food	$0.03	$0.03	$0.03	$0.03	$0.03	$0.03	$-
		Wages lost (self)	$0.18	$0.24	$0.11	$0.18	$0.24	$0.11	$-
		Wages lost (spouse)	$1.31	$1.44	$1.18	$1.31	$1.44	$1.18	$-
		Child care for other children	$0.07	$0.26	$(0.13)	$0.07	$0.26	$(0.13)	$-
		Transport	$0.08	$0.34	$(0.18)	$0.08	$0.34	$(0.18)	$-
		sub-total	*$1.66*	*$2.31*	*$1.01*	*$1.66*	*$2.31*	*$1.01*	*$-*
		ANC Visit 1	$1.66	$2.31	$1.01	$1.66	$2.31	$1.01	$-
		ANC Visit 2	$1.66	$2.31	$1.01	$1.66	$2.31	$1.01	$-

		ANC Visit 3	$1.66	$2.31	$1.01	$1.66	$2.31	$1.01	$-
		ANC Visit 4	$1.66	$2.31	$1.01	$1.66	$2.31	$1.01	$-
		Total ANC 1-4	*$6.64*	*$9.24*	*$4.04*	*$6.64*	*$9.24*	*$4.04*	*$-*
		ANC 4+ Gauteng	*$858,608.44*	*$912,433.05*	*$617,146.13*	*$548,555.39*	*$630,408.29*	*$384,808.76*	*$310,053.05*
		PNC Visit 1: Birth	$1.48	$2.07	$0.90	$1.48	$2.07	$0.90	$-
		PNC Visit 2: 6 week	$1.48	$2.07	$0.90	$1.48	$2.07	$0.90	$-
		PNC Visit 3: 10 week	$1.48	$2.07	$0.90	$1.48	$2.07	$0.90	$-
		PNC Visit 4: 14 week	$1.48	$2.07	$0.90	$1.48	$2.07	$0.90	$-
		PNC Visit 5: 9 months	$1.66	$2.31	$1.01	$1.66	$2.31	$1.01	$-
		Total PNC	*$7.60*	*$10.59*	*$4.61*	*$7.60*	*$10.59*	*$4.61*	*$-*
		PNC5+ (Fully immunized) Gauteng	*$1,296,345.38*	*$1,748,920.14*	*$811,585.05*	*$1,228,116.68*	*$1,596,840.13*	*$778,459.13*	*$68,228.70*
		Total users cost per ANC4+ & Fully immunized Gauteng	*$2,154,953.82*	*$2,661,353.19*	*$1,428,731.18*	*$1,776,672.07*	*$2,227,248.42*	*$1,163,267.89*	*$378,281.75*
	Annual program costs at maturity (year 5)								
	Implementation support								
		Development	$0.23	$0.28	$0.17				$0.23
		Start-up	$0.11	$0.14	$0.08				$0.11
		Training	$0.00	$0.00	$0.00				$0.00
		Personnel	$0.12	$0.15	$0.09				$0.12
		Buildings	$0.04	$0.04	$0.03				$0.04
		Transport	$0.02	$0.02	$0.01				$0.02
		Communication	$0.00	$0.00	$0.00				$0.00
		Sub-total implementation support	$0.51	$0.64	$0.39				$0.51
	Technology costs								
		Start-up/ Development	$0.00	$0.01	$0.00				$0.00
		Content maintenance	$0.05	$0.07	$0.04				$0.05
		Technology maintenance	$0.11	$0.14	$0.08				$0.11

		Project management/ personnel	$0.10	$0.12	$0.07				$0.10
		M&E	$0.00	$0.00	$0.00				$0.00
		Building/ Overhead	$0.07	$0.09	$0.06				$0.07
		Travel	$0.02	$0.02	$0.01				$0.02
		SMS text message delivery	$1.38	$2.36	$1.02				$1.38
		SMS text message translation	$0.01	$0.01	$0.01				
		Printing	$-	$-	$-				$-
		Sub-total technology	$1.74	$2.83	$1.30				$1.74
		Total program cost per user	$2.26	$3.47	$1.69				$2.26
		Total program cost Gauteng	*$405,491.14*	*$623,318.87*	*$303,252.74*				*$405,491.14*
		Total societal cost Gauteng	*$3,662,391.57*	*$4,676,987.41*	*$2,416,627.15*	*$2,633,741.65*	*$3,340,293.16*	*$1,687,274.35*	*$1,028,649.91*

[a]Utilization rates for ANC 2 have been inputted into LiST, which does not allow for differentiation between utilization of ANC 1-4; instead allowing for data on co-coverage of interventions or a flat coverage estimate to be inputted. The assumption in using ANC 2 is that the majority of clinical services in South Africa are provided within the first 2 ANC visits.

[b]Sub-national estimated adjusted based on sample population.

[c]MAMA: Mobile Alliance for Maternal Action;

[d]ANC: antenatal care;

[e]PNC: postnatal care
