# Supplementary material for: Forecasting the Value for Money of Mobile Maternal Health Information Messages on Improving Utilization of Maternal and Child Health Services in Gauteng, South Africa: Cost-Effectiveness Analysis
Source: JMIR Mhealth Uhealth. 2018 Jul 27;6(7):e153. doi: 10.2196/mhealth.8185 (PMC6086931; doi:10.2196/mhealth.8185)
Supplement: Multimedia Appendix 3 [file mhealth_v6i7e153_app3.pdf]

Year 1 Program costs in US $ for gradual rollout in Gauteng province, South Africa

	MAMA			Non-MAMA			Incremental
**Parameter**	**Base case**	**High**	**Low**	**Base case**	**High**	**Low**	
**Total users**							
Proportion ANC 4+	72%	85%	55%	46%	53%	38%	26%
Number ANC 4+ Gauteng	2,073	2,447	1,583	1,324	1,526	1,094	749
Proportion Fully immunized	95%	98%	92%	90%	94%	84%	5%
Number Fully immunized	2,735	2,821	2,649	2,591	2,706	2,418	144
Proportion ANC4+ & Fully immunized	67%	75%	57%	39%	53%	26%	
Number ANC4+ & Fully immunized	1,928.93	2,159.25	1,641.03	1,122.81	1,525.87	748.54	806
Incremental Lives Saved							190.00
Disability adjusted live years averted							5,130.00
**Provider costs**							
Peer educator time costs to register MAMA users	$ 0.08	$ 0.11	$ 0.04	-	-	-	
**Registration costs Gauteng**	**$ 221.40**	**$ 323.69**	**$ 124.22**				**$ 221.40**
ANC 1 Group counseling (5 minute peer educator)	$ 0.26	$ 0.37	$ 0.14	$ 0.26	$ 0.37	$ 0.14	
ANC 1 One on one consultation (10 minute Nurse time)	$ 1.03	$ 1.50	$ 0.58	$ 1.03	$ 1.50	$ 0.58	
**Total ANC1**	**$ 1.28**	**$ 1.87**	**$ 0.72**	**$ 1.28**	**$ 1.87**	**$ 0.72**	
**ANC 2**	$ 1.03	$ 1.50	$ 0.58	$ 1.03	$ 1.50	$ 0.58	
**ANC 3**	$ 1.03	$ 1.50	$ 0.58	$ 1.03	$ 1.50	$ 0.58	
**ANC 4**	$ 1.03	$ 1.50	$ 0.58	$ 1.03	$ 1.50	$ 0.58	
**Total ANC 4+**	**$ 4.36**	**$ 6.37**	**$ 2.45**	**$ 4.36**	**$ 6.37**	**$ 2.45**	
**ANC 4+ Gauteng**	**$ 9,033.29**	**$ 10,088.27**	**$ 5,983.43**	**$ 5,771.27**	**$ 6,970.08**	**$ 3,730.85**	**$ 3,262.02**

PNC 1 (10 minute Nurse time)	$ 1.03	$ 1.50	$ 0.58	$ 1.03	$ 1.50	$ 0.58	
PNC 2 (5 minute Nurse time)	$ 0.51	$ 0.75	$ 0.29	$ 0.51	$ 0.75	$ 0.29	
PNC 3 (5 minute Nurse time)	$ 0.51	$ 0.75	$ 0.29	$ 0.51	$ 0.75	$ 0.29	
PNC 4 (5 minute Nurse time)	$ 0.51	$ 0.75	$ 0.29	$ 0.51	$ 0.75	$ 0.29	
PNC 5 (5 minute Nurse time)	$ 0.51	$ 0.75	$ 0.29	$ 0.51	$ 0.75	$ 0.29	
**Total PNC 5**	**$ 3.08**	**$ 4.50**	**$ 1.73**	**$ 3.08**	**$ 4.50**	**$ 1.73**	
**PNC5+ (Fully immunized) Gauteng**	**$ 8,413.36**	**$ 11,911.72**	**$ 4,869.56**	**$ 7,970.55**	**$ 10,875.91**	**$ 4,670.81**	**$ 442.81**
**Total provider cost per ANC4+ & Fully immunized Gauteng**	**17,668**	**22,324**	**10,977**	**13,742**	**17,846**	**8,402**	**$ 3,926.23**
**Users' costs**							
**Mean PNC cost per person per visit**							
Food	$ 0.03	$ 0.03	$ 0.03	$ 0.03	$ 0.03	$ 0.03	$ -
Wages lost (self)	$ 0.18	$ 0.24	$ 0.11	$ 0.18	$ 0.24	$ 0.11	$ -
Wages lost (spouse)	$ 1.31	$ 1.44	$ 1.18	$ 1.31	$ 1.44	$ 1.18	$ -
Child care for other children	$ 0.07	$ 0.26	$ (0.13)	$ 0.07	$ 0.26	$ (0.13)	$ -
Transport	$ 0.08	$ 0.34	$ (0.18)	$ 0.08	$ 0.34	$ (0.18)	$ -
**sub-total PNC**	**$ 1.66**	**$ 2.31**	**$ 1.01**	**$ 1.66**	**$ 2.31**	**$ 1.01**	**$ -**
PNC Visit 1: Birth	$ 1.48	$ 2.07	$ 0.90	$ 1.48	$ 2.07	$ 0.90	$ -
PNC Visit 2: 6 week	$ 1.48	$ 2.07	$ 0.90	$ 1.48	$ 2.07	$ 0.90	$ -
PNC Visit 3: 10 week	$ 1.48	$ 2.07	$ 0.90	$ 1.48	$ 2.07	$ 0.90	$ -
PNC Visit 4: 14 week	$ 1.48	$ 2.07	$ 0.90	$ 1.48	$ 2.07	$ 0.90	$ -
PNC Visit 5: 9 months	$ 1.66	$ 2.31	$ 1.01	$ 1.66	$ 2.31	$ 1.01	$ -

**Total PNC**	**$ 7.60**	**$ 10.59**	**$ 4.61**	**$ 7.60**	**$ 10.59**	**$ 4.61**	$ -
**PNC5+ (Fully immunized) Gauteng**	**$ 20,784.94**	**$ 28,041.30**	**$ 13,012.54**	**$ 19,691.00**	**$ 25,602.92**	**$ 12,481.42**	**$ 1,093.94**
ANC Visit 1	$ 1.66	$ 2.31	$ 1.01	$ 1.66	$ 2.31	$ 1.01	$ -
ANC Visit 2	$ 1.66	$ 2.31	$ 1.01	$ 1.66	$ 2.31	$ 1.01	$ -
ANC Visit 3	$ 1.66	$ 2.31	$ 1.01	$ 1.66	$ 2.31	$ 1.01	$ -
ANC Visit 4	$ 1.66	$ 2.31	$ 1.01	$ 1.66	$ 2.31	$ 1.01	$ -
**Total ANC 1-4**	**$ 6.64**	**$ 9.24**	**$ 4.04**	**$ 6.64**	**$ 9.24**	**$ 4.04**	**$ -**
**ANC 4+ Gauteng**	**$ 13,766.49**	**$ 14,629.49**	**$ 9,895.01**	**$ 8,795.26**	**$ 10,107.65**	**$ 6,169.83**	**$ 4,971.23**
**Total users cost per ANC4+ & Fully immunized Gauteng**	**$ 34,551.44**	**$ 42,670.79**	**$ 22,907.55**	**$ 28,486.26**	**$ 35,710.57**	**$ 18,651.25**	**$ 6,065.18**
**Annual program costs: Year 4**							
**Implementation support**							
Development	$ 12.97	$ 16.22	$ 9.73				$ 12.97
Start-up	$ 6.17	$ 7.71	$ 4.63				$ 6.17
Training	$ 0.05	$ 0.07	$ 0.04				$ 0.05
Personnel	$ 6.38	$ 7.98	$ 4.79				$ 6.38
Buildings	$ 2.08	$ 2.59	$ 1.56				$ 2.08
Transport	$ 1.12	$ 1.40	$ 0.84				$ 1.12
Communication	$ 0.19	$ 0.23	$ 0.14				$ 0.19
Sub-total implementation support	$ 28.96	$ 36.20	$ 21.72				$ 28.96
**Technology costs**							
Start-up/ Development	$ 0.05	$ 0.41	$ 0.24				$ 0.05

Content maintenance	$ 3.64	$ 4.55	$ 2.73				$ 3.64
Technology maintenance	$ 2.88	$ 3.59	$ 2.16				$ 2.88
Project management/ personnel	$ 8.93	$ 11.16	$ 6.69				$ 8.93
M&E	$ 0.64	$ 0.80	$ 0.48				$ 0.64
Building/ Overhead	$ 3.50	$ 4.37	$ 2.62				$ 3.50
Travel	$ 3.94	$ 4.93	$ 2.96				$ 3.94
SMS Message delivery	$ 1.87	$ 3.19	$ 1.52				$ 1.87
SMS Translation	$ 0.60	$ 0.75	$ 0.45				$ 0.60
Printing	$ 1.64	$ 2.05	$ 1.23				$ 1.64
Sub-total technology	$ 27.68	$ 35.80	$ 21.09				$ 27.68
**Total program cost per user**	$ 56.65	$ 72.01	$ 42.81				$ 56.65
**Total program cost Gauteng**	**$ 163,082.27**	**$ 207,307.42**	**$ 123,250.26**				**$ 163,082.27**
**Total societal cost Gauteng**	**$ 215,301.76**	**$ 272,301.88**	**$ 157,135.03**	**$ 42,228.08**	**$ 53,556.56**	**$ 27,052.90**	**$ 173,073.69**
