# Supplementary material for: Forecasting the Value for Money of Mobile Maternal Health Information Messages on Improving Utilization of Maternal and Child Health Services in Gauteng, South Africa: Cost-Effectiveness Analysis
Source: JMIR Mhealth Uhealth. 2018 Jul 27;6(7):e153. doi: 10.2196/mhealth.8185 (PMC6086931; doi:10.2196/mhealth.8185)
Supplement: Multimedia Appendix 7 [file mhealth_v6i7e153_app4.pdf]

Year 2 Program costs in US $ for gradual rollout in Gauteng province, South Africa

	MAMA				Non-MAMA			Incremental
**Parameter**		**Base case**	**High**	**Low**	**Base case**	**High**	**Low**	
**Total users**								
Proportion ANC 4+		72%	85%	55%	46%	53%	38%	26%
Number ANC 4+ Gauteng		5,876	6,937	4,489	3,754	4,325	3,101	2,122
Proportion Fully immunized		95%	98%	92%	90%	94%	84%	5%
Number Fully immunized		7,753	7,998	7,508	7,345	7,671	6,855	408
Proportion ANC4+ & Fully immunized		67%	75%	57%	39%	53%	26%	
Number ANC4+ & Fully immunized		5,467.87	6,120.75	4,651.77	3,182.79	4,325.33	2,121.86	2,285
Incremental Lives Saved								190.00
Disability adjusted live years averted								5,130.00
**Provider costs**								
Peer educator time costs to register MAMA users	$	0.08	$ 0.11	$ 0.04	-	-	-	
**Registration costs Gauteng**	**$**	**627.61**	**$ 917.55**	**$ 352.13**				**$ 627.61**
ANC 1 Group counseling (5 minute peer educator)	$	0.26	$ 0.37	$ 0.14	$ 0.26	$ 0.37	$ 0.14	
ANC 1 One on one consultation (10 minute Nurse time)	$	1.03	$ 1.50	$ 0.58	$ 1.03	$ 1.50	$ 0.58	
**Total ANC1**	**$**	**1.28**	**$ 1.87**	**$ 0.72**	**$ 1.28**	**$ 1.87**	**$ 0.72**	
**ANC 2**	$	1.03	$ 1.50	$ 0.58	$ 1.03	$ 1.50	$ 0.58	
**ANC 3**	$	1.03	$ 1.50	$ 0.58	$ 1.03	$ 1.50	$ 0.58	
**ANC 4**	$	1.03	$ 1.50	$ 0.58	$ 1.03	$ 1.50	$ 0.58	
**Total ANC 4+**	**$**	**4.36**	**$ 6.37**	**$ 2.45**	**$ 4.36**	**$ 6.37**	**$ 2.45**	
**ANC 4+ Gauteng**	**$**	**25,606.35**	**$ 28,596.87**	**$ 16,961.03**	**$ 16,359.61**	**$ 19,757.84**	**$ 10,575.70**	**$ 9,246.74**

PNC 1 (10 minute Nurse time)	$	1.03	$ 1.50	$ 0.58	$ 1.03	$ 1.50	$ 0.58	
PNC 2 (5 minute Nurse time)	$	0.51	$ 0.75	$ 0.29	$ 0.51	$ 0.75	$ 0.29	
PNC 3 (5 minute Nurse time)	$	0.51	$ 0.75	$ 0.29	$ 0.51	$ 0.75	$ 0.29	
PNC 4 (5 minute Nurse time)	$	0.51	$ 0.75	$ 0.29	$ 0.51	$ 0.75	$ 0.29	
PNC 5 (5 minute Nurse time)	$	0.51	$ 0.75	$ 0.29	$ 0.51	$ 0.75	$ 0.29	
**Total PNC 5**	**$**	**3.08**	**$ 4.50**	**$ 1.73**	**$ 3.08**	**$ 4.50**	**$ 1.73**	
**PNC5+ (Fully immunized) Gauteng**	**$**	**23,849.05**	**$ 33,765.72**	**$ 13,803.58**	**$ 22,593.84**	**$ 30,829.57**	**$ 13,240.17**	**$ 1,255.21**
**Total provider cost per ANC4+ & Fully immunized Gauteng**		**50,083**	**63,280**	**31,117**	**38,953**	**50,587**	**23,816**	**$ 11,129.56**
**Users' costs**								
**Mean PNC cost per person per visit**								
Food	$	0.03	$ 0.03	$ 0.03	$ 0.03	$ 0.03	$ 0.03	$ -
Wages lost (self)	$	0.18	$ 0.24	$ 0.11	$ 0.18	$ 0.24	$ 0.11	$ -
Wages lost (spouse)	$	1.31	$ 1.44	$ 1.18	$ 1.31	$ 1.44	$ 1.18	$ -
Child care for other children	$	0.07	$ 0.26	$ (0.13)	$ 0.07	$ 0.26	$ (0.13)	$ -
Transport	$	0.08	$ 0.34	$ (0.18)	$ 0.08	$ 0.34	$ (0.18)	$ -
**sub-total PNC**	**$**	**1.66**	**$ 2.31**	**$ 1.01**	**$ 1.66**	**$ 2.31**	**$ 1.01**	**$ -**
PNC Visit 1: Birth	$	1.48	$ 2.07	$ 0.90	$ 1.48	$ 2.07	$ 0.90	$ -
PNC Visit 2: 6 week	$	1.48	$ 2.07	$ 0.90	$ 1.48	$ 2.07	$ 0.90	$ -
PNC Visit 3: 10 week	$	1.48	$ 2.07	$ 0.90	$ 1.48	$ 2.07	$ 0.90	$ -
PNC Visit 4: 14 week	$	1.48	$ 2.07	$ 0.90	$ 1.48	$ 2.07	$ 0.90	$ -
PNC Visit 5: 9 months	$	1.66	$ 2.31	$ 1.01	$ 1.66	$ 2.31	$ 1.01	$ -

**Total PNC**	**$ 7.60**	**$ 10.59**	**$ 4.61**	**$ 7.60**	**$ 10.59**	**$ 4.61**	$ -
**PNC5+ (Fully immunized) Gauteng**	**$ 58,918.35**	**$ 79,487.68**	**$ 36,886.20**	**$ 55,817.38**	**$ 72,575.71**	**$ 35,380.64**	**$ 3,100.97**
ANC Visit 1	$ 1.66	$ 2.31	$ 1.01	$ 1.66	$ 2.31	$ 1.01	$ -
ANC Visit 2	$ 1.66	$ 2.31	$ 1.01	$ 1.66	$ 2.31	$ 1.01	$ -
ANC Visit 3	$ 1.66	$ 2.31	$ 1.01	$ 1.66	$ 2.31	$ 1.01	$ -
ANC Visit 4	$ 1.66	$ 2.31	$ 1.01	$ 1.66	$ 2.31	$ 1.01	$ -
**Total ANC 1-4**	**$ 6.64**	**$ 9.24**	**$ 4.04**	**$ 6.64**	**$ 9.24**	**$ 4.04**	**$ -**
**ANC 4+ Gauteng**	**$ 39,023.39**	**$ 41,469.70**	**$ 28,049.03**	**$ 24,931.61**	**$ 28,651.79**	**$ 17,489.40**	**$ 14,091.78**
**Total users cost per ANC4+ & Fully immunized Gauteng**	**$ 97,941.74**	**$ 120,957.37**	**$ 64,935.23**	**$ 80,748.99**	**$ 101,227.50**	**$ 52,870.03**	**$ 17,192.75**
**Annual program costs: Year 4**							
**Implementation support**							
Development	$ 4.71	$ 5.72	$ 3.43				$ 4.71
Start-up	$ 2.24	$ 2.72	$ 1.63				$ 2.24
Training	$ -	$ -	$ -				$ -
Personnel	$ 2.30	$ 2.88	$ 1.73				$ 2.30
Buildings	$ 0.69	$ 0.86	$ 0.52				$ 0.69
Transport	$ 0.37	$ 0.47	$ 0.28				$ 0.37
Communication	$ 0.06	$ 0.08	$ 0.05				$ 0.06
Sub-total implementation support	$ 10.39	$ 12.73	$ 7.64				$ 10.39
**Technology costs**							
Start-up/ Development	$ 0.02	$ 0.14	$ 0.09				$ 0.02

Content maintenance	$ 1.58	$ 1.97	$ 1.18				$ 1.58
Technology maintenance	$ 4.52	$ 5.65	$ 3.39				$ 4.52
Project management/ personnel	$ 3.79	$ 4.74	$ 2.85				$ 3.79
M&E	$ 0.24	$ 0.30	$ 0.18				$ 0.24
Building/ Overhead	$ 1.60	$ 2.00	$ 1.20				$ 1.60
Travel	$ 1.05	$ 1.32	$ 0.79				$ 1.05
SMS Message delivery	$ 1.21	$ 2.36	$ 1.02				$ 1.21
SMS Translation	$ 0.23	$ 0.28	$ 0.17				
Printing	$ -	$ -	$ -				$ -
Sub-total technology	$ 14.24	$ 18.77	$ 10.87				$ 14.24
**Total program cost per user**	$ 24.63	$ 31.50	$ 18.51				$ 24.63
**Total program cost Gauteng**	**$ 200,979.94**	**$ 257,107.75**	**$ 151,049.61**				**$ 200,979.94**
**Total societal cost Gauteng**	**$ 349,004.68**	**$ 441,345.26**	**$ 247,101.58**	**$ 119,702.44**	**$ 151,814.91**	**$ 76,685.90**	**$ 229,302.24**
