# Supplementary material for: Forecasting the Value for Money of Mobile Maternal Health Information Messages on Improving Utilization of Maternal and Child Health Services in Gauteng, South Africa: Cost-Effectiveness Analysis
Source: JMIR Mhealth Uhealth. 2018 Jul 27;6(7):e153. doi: 10.2196/mhealth.8185 (PMC6086931; doi:10.2196/mhealth.8185)
Supplement: Multimedia Appendix 5 [file mhealth_v6i7e153_app5.pdf]

**Year 3 Program costs in US $ for gradual rollout in Gauteng province, South Africa**

| | MAMA | | | Non-MAMA | | | Incremental |
|---|---|---|---|---|---|---|---|
| **Parameter** | **Base case** | **High** | **Low** | **Base case** | **High** | **Low** | |
| **Total users** | | | | | | | |
| Proportion ANC 4+ | 72% | 85% | 55% | 46% | 53% | 38% | 26% |
| Number ANC 4+ Gauteng | 13,261 | 15,656 | 10,130 | 8,473 | 9,762 | 6,999 | 4,789 |
| Proportion Fully immunized | 95% | 98% | 92% | 90% | 94% | 84% | 5% |
| Number Fully immunized | 17,498 | 18,050 | 16,945 | 16,577 | 17,313 | 15,472 | 921 |
| Proportion ANC4+ & Fully immunized | 67% | 75% | 57% | 39% | 53% | 26% | |
| Number ANC4+ & Fully immunized | 12,340.46 | 13,813.95 | 10,498.60 | 7,183.25 | 9,761.86 | 4,788.84 | 5,157 |
| Incremental Lives Saved | | | | | | | 190.00 |
| Disability adjusted live years averted | | | | | | | 5,130.00 |
| **Provider costs** | | | | | | | |
| Peer educator time costs to register MAMA users | $ 0.08 | $ 0.11 | $ 0.04 | - | - | - | |
| **Registration costs Gauteng** | **$ 1,416.45** | **$ 2,070.82** | **$ 794.73** | | | | **$ 1,416.45** |
| ANC 1 Group counseling (5 minute peer educator) | $ 0.26 | $ 0.37 | $ 0.14 | $ 0.26 | $ 0.37 | $ 0.14 | |
| ANC 1 One on one consultation (10 minute Nurse time) | $ 1.03 | $ 1.50 | $ 0.58 | $ 1.03 | $ 1.50 | $ 0.58 | |
| **Total ANC1** | **$ 1.28** | **$ 1.87** | **$ 0.72** | **$ 1.28** | **$ 1.87** | **$ 0.72** | |
| **ANC 2** | $ 1.03 | $ 1.50 | $ 0.58 | $ 1.03 | $ 1.50 | $ 0.58 | |
| **ANC 3** | $ 1.03 | $ 1.50 | $ 0.58 | $ 1.03 | $ 1.50 | $ 0.58 | |
| **ANC 4** | $ 1.03 | $ 1.50 | $ 0.58 | $ 1.03 | $ 1.50 | $ 0.58 | |
| **Total ANC 4+** | **$ 4.36** | **$ 6.37** | **$ 2.45** | **$ 4.36** | **$ 6.37** | **$ 2.45** | |
| **ANC 4+ Gauteng** | **$ 57,791.10** | **$ 64,540.43** | **$ 38,279.44** | **$ 36,922.09** | **$ 44,591.57** | **$ 23,868.36** | **$ 20,869.01** |

| | | | | | | | |
|---|---|---|---|---|---|---|---|
| PNC 1 (10 minute Nurse time) | $ 1.03 | $ 1.50 | $ 0.58 | $ 1.03 | $ 1.50 | $ 0.58 | |
| PNC 2 (5 minute Nurse time) | $ 0.51 | $ 0.75 | $ 0.29 | $ 0.51 | $ 0.75 | $ 0.29 | |
| PNC 3 (5 minute Nurse time) | $ 0.51 | $ 0.75 | $ 0.29 | $ 0.51 | $ 0.75 | $ 0.29 | |
| PNC 4 (5 minute Nurse time) | $ 0.51 | $ 0.75 | $ 0.29 | $ 0.51 | $ 0.75 | $ 0.29 | |
| PNC 5 (5 minute Nurse time) | $ 0.51 | $ 0.75 | $ 0.29 | $ 0.51 | $ 0.75 | $ 0.29 | |
| **Total PNC 5** | **$ 3.08** | **$ 4.50** | **$ 1.73** | **$ 3.08** | **$ 4.50** | **$ 1.73** | |
| **PNC5+ (Fully immunized) Gauteng** | **$ 53,825.04** | **$ 76,206.03** | **$ 31,153.37** | **$ 50,992.15** | **$ 69,579.41** | **$ 29,881.80** | **$ 2,832.90** |
| **Total provider cost per ANC4+ & Fully immunized Gauteng** | **113,033** | **142,817** | **70,228** | **87,914** | **114,171** | **53,750** | **$ 25,118.35** |
| **Users' costs** | | | | | | | |
| **Mean PNC cost per person per visit** | | | | | | | |
| Food | $ 0.03 | $ 0.03 | $ 0.03 | $ 0.03 | $ 0.03 | $ 0.03 | $ - |
| Wages lost (self) | $ 0.18 | $ 0.24 | $ 0.11 | $ 0.18 | $ 0.24 | $ 0.11 | $ - |
| Wages lost (spouse) | $ 1.31 | $ 1.44 | $ 1.18 | $ 1.31 | $ 1.44 | $ 1.18 | $ - |
| Child care for other children | $ 0.07 | $ 0.26 | $ (0.13) | $ 0.07 | $ 0.26 | $ (0.13) | $ - |
| Transport | $ 0.08 | $ 0.34 | $ (0.18) | $ 0.08 | $ 0.34 | $ (0.18) | $ - |
| **sub-total PNC** | **$ 1.66** | **$ 2.31** | **$ 1.01** | **$ 1.66** | **$ 2.31** | **$ 1.01** | **$ -** |
| PNC Visit 1: Birth | $ 1.48 | $ 2.07 | $ 0.90 | $ 1.48 | $ 2.07 | $ 0.90 | $ - |
| PNC Visit 2: 6 week | $ 1.48 | $ 2.07 | $ 0.90 | $ 1.48 | $ 2.07 | $ 0.90 | $ - |
| PNC Visit 3: 10 week | $ 1.48 | $ 2.07 | $ 0.90 | $ 1.48 | $ 2.07 | $ 0.90 | $ - |
| PNC Visit 4: 14 week | $ 1.48 | $ 2.07 | $ 0.90 | $ 1.48 | $ 2.07 | $ 0.90 | $ - |
| PNC Visit 5: 9 months | $ 1.66 | $ 2.31 | $ 1.01 | $ 1.66 | $ 2.31 | $ 1.01 | $ - |

| | | | | | | | |
|---|---|---|---|---|---|---|---|
| **Total PNC** | **$ 7.60** | **$ 10.59** | **$ 4.61** | **$ 7.60** | **$ 10.59** | **$ 4.61** | $ - |
| **PNC5+ (Fully immunized) Gauteng** | **$ 132,973.12** | **$ 179,396.14** | **$ 83,248.64** | **$ 125,974.53** | **$ 163,796.48** | **$ 79,850.74** | **$ 6,998.59** |
| ANC Visit 1 | $ 1.66 | $ 2.31 | $ 1.01 | $ 1.66 | $ 2.31 | $ 1.01 | $ - |
| ANC Visit 2 | $ 1.66 | $ 2.31 | $ 1.01 | $ 1.66 | $ 2.31 | $ 1.01 | $ - |
| ANC Visit 3 | $ 1.66 | $ 2.31 | $ 1.01 | $ 1.66 | $ 2.31 | $ 1.01 | $ - |
| ANC Visit 4 | $ 1.66 | $ 2.31 | $ 1.01 | $ 1.66 | $ 2.31 | $ 1.01 | $ - |
| **Total ANC 1-4** | **$ 6.64** | **$ 9.24** | **$ 4.04** | **$ 6.64** | **$ 9.24** | **$ 4.04** | **$ -** |
| **ANC 4+ Gauteng** | **$ 88,072.08** | **$ 93,593.16** | **$ 63,304.00** | **$ 56,268.28** | **$ 64,664.37** | **$ 39,471.90** | **$ 31,803.81** |
| **Total users cost per ANC4+ & Fully immunized Gauteng** | **$ 221,045.20** | **$ 272,989.31** | **$ 146,552.64** | **$ 182,242.81** | **$ 228,460.85** | **$ 119,322.64** | **$ 38,802.39** |
| **Annual program costs: Year 4** | | | | | | | |
| **Implementation support** | | | | | | | |
| Development | $ 2.15 | $ 2.61 | $ 1.57 | | | | $ 2.15 |
| Start-up | $ 1.02 | $ 1.24 | $ 0.75 | | | | $ 1.02 |
| Training | $ 0.00 | $ 0.01 | $ 0.00 | | | | $ 0.00 |
| Personnel | $ 1.07 | $ 1.33 | $ 0.80 | | | | $ 1.07 |
| Buildings | $ 0.32 | $ 0.40 | $ 0.24 | | | | $ 0.32 |
| Transport | $ 0.17 | $ 0.22 | $ 0.13 | | | | $ 0.17 |
| Communication | $ 0.03 | $ 0.04 | $ 0.02 | | | | $ 0.03 |
| ***Sub-total implementation support*** | $ 4.67 | $ 5.84 | $ 3.51 | | | | $ 4.67 |
| **Technology costs** | | | | | | | |
| Start-up/ Development | $ 0.01 | $ 0.07 | $ 0.04 | | | | $ 0.01 |

| | | | | | | | |
|---|---|---|---|---|---|---|---|
| Content maintenance | $ 0.50 | $ 0.62 | $ 0.37 | | | | $ 0.50 |
| Technology maintenance | $ 1.00 | $ 1.26 | $ 0.75 | | | | $ 1.00 |
| Project management/ personnel | $ 0.85 | $ 1.06 | $ 0.63 | | | | $ 0.85 |
| M&E | $ 0.01 | $ 0.01 | $ 0.00 | | | | $ 0.01 |
| Building/ Overhead | $ 0.66 | $ 0.83 | $ 0.50 | | | | $ 0.66 |
| Travel | $ 0.17 | $ 0.21 | $ 0.13 | | | | $ 0.17 |
| SMS Message delivery | $ 1.26 | $ 2.36 | $ 1.02 | | | | $ 1.26 |
| SMS Translation | $ 0.10 | $ 0.13 | $ 0.08 | | | | |
| Printing | $ - | $ - | $ - | | | | $ - |
| ***Sub-total technology*** | $ 4.56 | $ 6.54 | $ 3.53 | | | | $ 4.56 |
| **Total program cost per user** | $ 9.23 | $ 12.38 | $ 7.03 | | | | $ 9.23 |
| **Total program cost Gauteng** | **$ 170,007.86** | **$ 228,013.02** | **$ 129,551.78** | | | | **$ 170,007.86** |
| **Total societal cost Gauteng** | **$ 504,085.64** | **$ 643,819.60** | **$ 346,331.95** | **$ 270,157.04** | **$ 342,631.83** | **$ 173,072.80** | **$ 233,928.60** |
