# Supplementary material for: Forecasting the Value for Money of Mobile Maternal Health Information Messages on Improving Utilization of Maternal and Child Health Services in Gauteng, South Africa: Cost-Effectiveness Analysis
Source: JMIR Mhealth Uhealth. 2018 Jul 27;6(7):e153. doi: 10.2196/mhealth.8185 (PMC6086931; doi:10.2196/mhealth.8185)
Supplement: Multimedia Appendix 6 [file mhealth_v6i7e153_app6.pdf]

Year 4 Program costs in US $ for gradual rollout in Gauteng province, South Africa

	MAMA			Non-MAMA			Incremental
**Parameter**	**Base case**	**High**	**Low**	**Base case**	**High**	**Low**	
**Total users**							
Proportion ANC 4+	72%	85%	55%	46%	53%	38%	26%
Number ANC 4+ Gauteng	41,193.99	48,632	31,468	26,318	30,323	21,741	14,876
Proportion Fully immunized	95%	98%	92%	90%	94%	84%	5%
Number Fully immunized	54,353	56,070	52,637	51,492	53,781	48,060	2,861
Proportion ANC4+ & Fully immunized	67%	75%	57%	39%	53%	26%	
Number ANC4+ & Fully immunized	38,333.29	42,910.40	32,611.91	22,313.41	30,323.35	14,875.61	16,020
Incremental Lives Saved							190.00
Disability adjusted live years averted							5,130.00
**Provider costs**							
Peer educator time costs to register MAMA users	$ 0.08	$ 0.11	$ 0.04	-	-	-	
**Registration costs Gauteng**	**$ 4,399.93**	**$ 6,432.59**	**$ 2,468.67**				**$ 4,399.93**
ANC 1 Group counseling (5 minute peer educator)	$ 0.26	$ 0.37	$ 0.14	$ 0.26	$ 0.37	$ 0.14	
ANC 1 One on one consultation (10 minute Nurse time)	$ 1.03	$ 1.50	$ 0.58	$ 1.03	$ 1.50	$ 0.58	
**Total ANC1**	**$ 1.28**	**$ 1.87**	**$ 0.72**	**$ 1.28**	**$ 1.87**	**$ 0.72**	
**ANC 2**	$ 1.03	$ 1.50	$ 0.58	$ 1.03	$ 1.50	$ 0.58	
**ANC 3**	$ 1.03	$ 1.50	$ 0.58	$ 1.03	$ 1.50	$ 0.58	
**ANC 4**	$ 1.03	$ 1.50	$ 0.58	$ 1.03	$ 1.50	$ 0.58	
**Total ANC 4+**	**$ 4.36**	**$ 6.37**	**$ 2.45**	**$ 4.36**	**$ 6.37**	**$ 2.45**	
**ANC 4+ Gauteng**	**$ 179,517.01**	**$ 200,482.52**	**$ 118,907.77**	**$ 114,691.42**	**$ 138,515.19**	**$ 74,142.49**	**$ 64,825.59**

PNC 1 (10 minute Nurse time)	$ 1.03	$ 1.50	$ 0.58	$ 1.03	$ 1.50	$ 0.58	
PNC 2 (5 minute Nurse time)	$ 0.51	$ 0.75	$ 0.29	$ 0.51	$ 0.75	$ 0.29	
PNC 3 (5 minute Nurse time)	$ 0.51	$ 0.75	$ 0.29	$ 0.51	$ 0.75	$ 0.29	
PNC 4 (5 minute Nurse time)	$ 0.51	$ 0.75	$ 0.29	$ 0.51	$ 0.75	$ 0.29	
PNC 5 (5 minute Nurse time)	$ 0.51	$ 0.75	$ 0.29	$ 0.51	$ 0.75	$ 0.29	
**Total PNC 5**	**$ 3.08**	**$ 4.50**	**$ 1.73**	**$ 3.08**	**$ 4.50**	**$ 1.73**	
**PNC5+ (Fully immunized) Gauteng**	**$ 167,197.21**	**$ 236,719.46**	**$ 96,771.99**	**$ 158,397.36**	**$ 216,135.16**	**$ 92,822.12**	**$ 8,799.85**
**Total provider cost per ANC4+ & Fully immunized Gauteng**	**351,114**	**443,635**	**218,148**	**273,089**	**354,650**	**166,965**	**$ 78,025.37**
**Users' costs**							
**Mean PNC cost per person per visit**							
Food	$ 0.03	$ 0.03	$ 0.03	$ 0.03	$ 0.03	$ 0.03	$ -
Wages lost (self)	$ 0.18	$ 0.24	$ 0.11	$ 0.18	$ 0.24	$ 0.11	$ -
Wages lost (spouse)	$ 1.31	$ 1.44	$ 1.18	$ 1.31	$ 1.44	$ 1.18	$ -
Child care for other children	$ 0.07	$ 0.26	$ (0.13)	$ 0.07	$ 0.26	$ (0.13)	$ -
Transport	$ 0.08	$ 0.34	$ (0.18)	$ 0.08	$ 0.34	$ (0.18)	$ -
**sub-total PNC**	**$ 1.66**	**$ 2.31**	**$ 1.01**	**$ 1.66**	**$ 2.31**	**$ 1.01**	**$ -**
PNC Visit 1: Birth	$ 1.48	$ 2.07	$ 0.90	$ 1.48	$ 2.07	$ 0.90	$ -
PNC Visit 2: 6 week	$ 1.48	$ 2.07	$ 0.90	$ 1.48	$ 2.07	$ 0.90	$ -
PNC Visit 3: 10 week	$ 1.48	$ 2.07	$ 0.90	$ 1.48	$ 2.07	$ 0.90	$ -
PNC Visit 4: 14 week	$ 1.48	$ 2.07	$ 0.90	$ 1.48	$ 2.07	$ 0.90	$ -
PNC Visit 5: 9 months	$ 1.66	$ 2.31	$ 1.01	$ 1.66	$ 2.31	$ 1.01	$ -

**Total PNC**	**$ 7.60**	**$ 10.59**	**$ 4.61**	**$ 7.60**	**$ 10.59**	**$ 4.61**	$ -
**PNC5+ (Fully immunized) Gauteng**	**$ 413,055.59**	**$ 557,259.86**	**$ 258,596.01**	**$ 391,315.82**	**$ 508,802.48**	**$ 248,041.07**	**$ 21,739.77**
ANC Visit 1	$ 1.66	$ 2.31	$ 1.01	$ 1.66	$ 2.31	$ 1.01	$ -
ANC Visit 2	$ 1.66	$ 2.31	$ 1.01	$ 1.66	$ 2.31	$ 1.01	$ -
ANC Visit 3	$ 1.66	$ 2.31	$ 1.01	$ 1.66	$ 2.31	$ 1.01	$ -
ANC Visit 4	$ 1.66	$ 2.31	$ 1.01	$ 1.66	$ 2.31	$ 1.01	$ -
**Total ANC 1-4**	**$ 6.64**	**$ 9.24**	**$ 4.04**	**$ 6.64**	**$ 9.24**	**$ 4.04**	**$ -**
**ANC 4+ Gauteng**	**$ 273,579.11**	**$ 290,729.29**	**$ 196,641.78**	**$ 174,786.65**	**$ 200,867.51**	**$ 122,611.93**	**$ 98,792.46**
**Total users cost per ANC4+ & Fully immunized Gauteng**	**$ 686,634.70**	**$ 847,989.14**	**$ 455,237.78**	**$ 566,102.47**	**$ 709,669.98**	**$ 370,653.00**	**$ 120,532.22**
**Annual program costs: Year 4**							
**Implementation support**							
Development	$ 0.69	$ 0.87	$ 0.52				$ 0.69
Start-up	$ 0.33	$ 0.41	$ 0.25				$ 0.33
Training	$ -	$ -	$ -				$ -
Personnel	$ 0.36	$ 0.45	$ 0.27				$ 0.36
Buildings	$ 0.11	$ 0.13	$ 0.08				$ 0.11
Transport	$ 0.06	$ 0.07	$ 0.04				$ 0.06
Communication	$ 0.01	$ 0.01	$ 0.01				$ 0.01
Sub-total implementation support	$ 1.56	$ 1.95	$ 1.17				$ 1.56
**Technology costs**							
Start-up/ Development	$ 0.00	$ 0.02	$ 0.01				$ 0.00

Content maintenance	$ 0.17	$ 0.21	$ 0.12				$ 0.17
Technology maintenance	$ 0.34	$ 0.42	$ 0.25				$ 0.34
Project management/ personnel	$ 0.29	$ 0.36	$ 0.22				$ 0.29
M&E	$ 0.00	$ 0.00	$ 0.00				$ 0.00
Building/ Overhead	$ 0.22	$ 0.28	$ 0.17				$ 0.22
Travel	$ 0.06	$ 0.07	$ 0.04				$ 0.06
SMS Message delivery	$ 1.32	$ 2.36	$ 1.02				$ 1.32
SMS Translation	$ 0.03	$ 0.04	$ 0.02				
Printing	$ -	$ -	$ -				$ -
Sub-total technology	$ 2.43	$ 3.77	$ 1.87				$ 2.43
**Total program cost per user**	$ 3.98	$ 5.71	$ 3.03				$ 3.98
**Total program cost Gauteng**	**$ 227,964.40**	**$ 326,872.13**	**$ 173,583.78**				**$ 227,964.40**
**Total societal cost Gauteng**	**$ 1,265,713.25**	**$ 1,618,495.85**	**$ 846,970.00**	**$ 839,191.26**	**$1,064,320.34**	**$ 537,617.61**	**$ 426,521.99**
